# Correction to: Selective role of the translin/trax RNase complex in hippocampal synaptic plasticity

**DOI:** 10.1186/s13041-021-00761-2

**Published:** 2021-03-05

**Authors:** Alan Jung Park, Mahesh Shivarama Shetty, Jay M. Baraban, Ted Abel

**Affiliations:** 1 Department of Biology, University of Pennsylvania, Philadelphia, PA USA; 2 Department of Neuroscience and Pharmacology, Carver College of Medicine, University of Iowa, 2-471 Bowen Science Building, 51 Newton Road, Iowa, IA 52242 USA; 3 Iowa Neuroscience Institute, Carver College of Medicine, University of Iowa, 2312 Pappajohn Biomedical Discovery Building, 169 Newton Road, Iowa, IA 52242 USA; 4 The Solomon H. Snyder Department of Neuroscience, Johns Hopkins University School of Medicine, Baltimore, MD USA; 5 Gogos Lab, Mortimer B. Zuckerman Mind Brain Behavior Institute, Jerome L. Greene Science Center, Columbia University, L5-053, 3227 Broadway, New York, NY 0027 USA

## Correction to: Mol Brain (2020) 13:145. https://doi.org/10.1186/s13041-020-00691-5

Following publication of the original article [1], the authors identified an error in Fig. 3a and its caption.

The concentration of DHPG was incorrectly given as 100 mM instead of 100 μM in Fig. 3a and its caption. The original article has been updated to correct this.
